# The rhetorician's craft, distinctions in science, and political morality

**DOI:** 10.1186/1747-5341-1-7

**Published:** 2006-05-02

**Authors:** John Z Sadler

**Affiliations:** 1 Department of Psychiatry, The University of Texas Southwestern Medical Center at Dallas, 5323 Harry Hines Boulevard, Dallas, TX 75390-9070, USA

## Abstract

In his response to Szasz' Secular Humanism and Scientific Psychiatry, the author considers the use of rhetorical devices in Szasz' work, Szasz' avoidance of acknowledging psychiatry's scientific distinctions, and Szaszian libertarianism versus liberalism.

## 

Thomas Szasz seems like an old friend to me, though we've never met, having only corresponded over e-mail about his work appearing in *Philosophy, Psychiatry, & Psychology*. I read *The Myth of Mental Illness *late in high school (early 1970's for me) and found myself fascinated by his passion, his close observations of social processes, and his ability to persuade. I read most of my Szasz in a time when the counterculture had questioned authority extensively, Marxism was respectable for many American youth, coeds wore Mao or Che t-shirts, and Ken Kesey had poisoned the psychiatric well for many of my pre-med peers with *One Flew Over the Cuckoo's Nest*. I took away from *Myth*, and many of Szasz' other works, an appreciation for the profound social power held by psychiatry, and a wariness about its potential for abuse. This was thirty-five years ago.

Today is different from the 1970's. Today American college kids both celebrate diversity (multiculturalism is everywhere) and fear it (they isolate themselves in virtual groupthink communities), psychiatry is more biomedical (and perhaps a little less controversial) than ever, and the American socially marginal (the poor, the retarded, the criminal, the "mentally ill") may well be worse off than in any other time in my life. So it is with a feeling of sadness that I comment on my "old friend's" work today. This current essay of Szasz' betrays little in the way of development of his ideas compared to his thought of thirty-five years ago, despite having a dramatically different social world and a different psychiatry. I can, however, salute his steadfastness, resolve, and energy, which I hope to possess if I'm at it for as long as Szasz has been.

I have three general and short points to make in response to Szasz' essay. The first point concerns the craft of rhetoric or polemic, the second psychiatry's distinction in science, and the third concerns political morality.

I believe readers of this journal or any essay should be aware of the craft of polemic – how one makes one's point persuasive and wins others over. I cannot say that I am a student of this field, but I have a practiced sense of the technique of polemic from my experiences as a journal editor. Dr. Szasz is peerless in this craft. We can appreciate the rhetorical techniques in this very essay. To mention a few: (1) The emotional tenor of the essay is brisk, high-key, exuberant, it shouts its own importance. (2) Value-laden adjectives undercut the credibility of contrary viewpoints: "this *ostensibly *medical specialty", *"so-called' *psychiatric drugs' *miraculously *appeared" for instance. (3) What might be called the "duped-public technique" applies to this phrase: "Politicians and the public quickly accepted the psychiatrists' claim . . .". The technique implies 'we' know better than those foolish others. (4) Overstatement:". . . mental illnesses are brain diseases effectively treated with drugs – became *dogma*, and deviation from it *heresy." *(5) Overgeneralization: *"Most idle, indigent, unwanted *persons continue to be incarcerated in mental hospitals – . . ." (6) Distortion of the truth: "We are proud of our criminal justice system because it protects the accused from the power of the state, a power we distrust because *its avowed aim is to harm the individual*. (All italics mine.) To name a few examples – many more can be found in this PEHM essay. Readers should check my essay for these techniques as well. These techniques build an argument, but, in my opinion, do not help us understand each other nor bring us closer to the truth or the good.

My second point can be made quickly and simply. After all these decades of the development of the sciences of psychiatry, involving hundreds of millions of competitively-won federal grant dollars, tens of thousands of peer-reviewed publications in the most distinguished scientific journals, worldwide accolades for psychiatric scientists, and widely replicated demonstrations of biological abnormalities from the molecular to the anatomical level, Dr. Szasz still maintains there is no disease in mental illness. He must believe that not only are the public and politicians duped, but also non-psychiatric scientists, science journal editors, the U.S. National Academy of Sciences nominating committees, as well as the Nobel Prize nominating committees. This colossal level of duping of the world's smartest people I find very hard to believe.

Szasz' latest book, *Faith in Freedom: Libertarian Principles and Psychiatric Practices*, (which I have not yet read), does suggest his ongoing commitment to a libertarian political philosophy, which I cannot support. As far as the politics of psychiatry go, for a Szaszian libertarian, of crucial concern is civil liberties, hence Szasz' prevailing concern about psychiatric coercions. But civil liberties have their limits as a political value. All political systems, even libertarianism, have coercive elements. Indeed, the coercive elements of political society is the tradeoff for social goods: I may not like prisons, mental hospitals, stop signs, taxes, requirements to go to school, or automobile emission controls (for example) because they infringe on my personal freedoms, but I am willing to suffer these coercions in order to enjoy the corresponding, and substantial, benefits they offer. Indeed, they represent the public-policy result of "consensual relations" deliberated upon and freely chosen in our society. As a liberal I also put a great value on civil liberties, and respect for them is shared by libertarians and liberals. Where the libertarian and the liberal differ is in concern for those who have not been lucky in the "natural lottery" – those who are poor, or ignorant, or impaired, or deviant for reasons outside of their own control. The liberal believes that the good society should provide mechanisms for aiding these people, while simultaneously supporting and encouraging achievement by the endowed. Where the libertarian and liberal disagree, and a place where I suspect Szasz and Sadler disagree, is (1) who qualifies as the poorly endowed and is deserving of aid, (2) the relative weights of civil liberties versus aid for the poorly endowed, and perhaps, (3) how and to what degree the latter two sets of values can be balanced for the good of all. I don't believe that whatever you call them, all people living in cardboard boxes under the expressway should be simply left to die in the cold, their civil liberties preserved. There must be a better political solution than that.

## Competing interests

The author(s) declare that they have no competing interests.

